# Examining the stability of dementia‐relevant speech measures in a high‐frequency picture description task repeated over 5 days

**DOI:** 10.1002/alz70856_100541

**Published:** 2025-12-25

**Authors:** Jessica Robin, Rosanne L. van den Berg, Mengdan Xu, Michael J. Spilka, Francesca K Cormack, Roos J Jutten, Casper de Boer, Sietske A.M Sikkes

**Affiliations:** ^1^ Cambridge Cognition, Cambridge, United Kingdom; ^2^ Alzheimer Center Amsterdam, Neurology, Vrije Universiteit Amsterdam, Amsterdam UMC location VUmc, Amsterdam, Netherlands; ^3^ Cambridge Cognition, Toronto, ON, Canada

## Abstract

**Background:**

Speech characteristics including pause rate, noun/pronoun use and information content vary in mild cognitive impairment (MCI) and in dementia due to Alzheimer's disease (AD). The picture description task can be used to elicit naturalistic speech samples to measure these changes. This short, low‐burden task is conducive to remote, higher‐frequency data collection but it is unknown how reliable speech measures are over repeated testing. We leveraged data from a 5‐day testing session with repeated and alternating pictures on each day to test how speech characteristics vary in cognitively unimpaired memory clinic patients.

**Method:**

50 cognitively unimpaired Dutch‐speaking adults (29F/21M; mean age = 68.4, SD = 6.2 years; mean education = 15.3, SD = 3.8 years) completed a 5‐day burst testing session with two picture description tasks each day. In the repeated condition, the same picture stimulus was shown; in the alternating condition, a unique picture was shown each day. We tested selected speech measures for differences between subsequent test sessions by calculating mean paired differences and their 95% confidence intervals (95%CI) and using intra‐class correlations (ICCs) to assess reliability over the five days of testing.

**Result:**

The mean paired difference between subsequent testing days was not different from zero (95%CI) for most speech measures, with more variability for the alternating picture. In both conditions, positive mean paired differences indicated a pattern to produce more words in subsequent days of testing. Test‐retest reliability across the five sessions ranged from ICC = 0.22‐0.92, with 7/10 selected measures having ICC > 0.5 in both conditions. The highest ICCs were obtained by averaging the speech measures across the two picture conditions.

**Conclusion:**

Speech patterns appear to be stable and reliable in high‐frequency administration of the picture description task. Repeated administration may entail learning and/or test familiarity effects as evidenced by longer descriptions throughout testing. Averaging the two pictures led to the highest reliability estimates, suggesting that multiple administrations of the task may be optimal. This research demonstrates that picture description is an ecologically valid, low burden task suitable for remote, higher‐frequency, longitudinal testing. Future work will probe how these effects carry over to MCI and AD populations.

